# The effect of immigration policy reform on mental health in people from minoritised ethnic groups in England: an interrupted time series analysis of longitudinal data from the UK Household Longitudinal Study cohort

**DOI:** 10.1016/S2215-0366(23)00412-1

**Published:** 2024-03

**Authors:** Annie Jeffery, Connor Gascoigne, Jennifer Dykxhoorn, Marta Blangiardo, Sara Geneletti, Gianluca Baio, James B Kirkbride

**Affiliations:** a Division of Psychiatry, University College London, London, UK; b Department of Statistical Science, University College London, London, UK; c Department of Epidemiology and Biostatistics, Imperial College London, London, UK; d Department of Statistics, London School of Economics and Political Science, London, UK

## Abstract

**Background:**

In 2012, the UK Government announced a series of immigration policy reforms known as the hostile environment policy, culminating in the Windrush scandal. We aimed to investigate the effect of the hostile environment policy on mental health for people from minoritised ethnic backgrounds. We hypothesised that people from Black Caribbean backgrounds would have worse mental health relative to people from White ethnic backgrounds after the Immigration Act 2014 and the Windrush scandal media coverage in 2017, since they were particularly targeted.

**Methods:**

Using data from the UK Household Longitudinal Study, we performed a Bayesian interrupted time series analysis, accounting for fixed effects of confounders (sex, age, urbanicity, relationship status, number of children, education, physical or mental health impairment, housing, deprivation, employment, place of birth, income, and time), and random effects for residual temporal and spatial variation. We measured mental ill health using a widely used, self-administered questionnaire on psychological distress, the 12-item General Health Questionnaire (GHQ-12). We compared mean differences (MDs) and 95% credible intervals (CrIs) in mental ill health among people from minoritised ethnic groups (Black Caribbean, Black African, Indian, Bangladeshi, and Pakistani) relative to people of White ethnicity during three time periods: before the Immigration Act 2014, after the Immigration Act 2014, and after the start of the Windrush scandal media coverage in 2017.

**Findings:**

We included 58 087 participants with a mean age of 45·0 years (SD 34·6; range 16–106), including 31 168 (53·6%) female and 26 919 (46·3%) male participants. The cohort consisted of individuals from the following ethnic backgrounds: 2519 (4·3%) Black African, 2197 (3·8%) Black Caribbean, 3153 (5·4%) Indian, 1584 (2·7%) Bangladeshi, 2801 (4·8%) Pakistani, and 45 833 (78·9%) White. People from Black Caribbean backgrounds had worse mental health than people of White ethnicity after the Immigration Act 2014 (MD in GHQ-12 score 0·67 [95% CrI 0·06–1·28]) and after the 2017 media coverage (1·28 [0·34–2·21]). For Black Caribbean participants born outside of the UK, mental health worsened after the Immigration Act 2014 (1·25 [0·11–2·38]), and for those born in the UK, mental health worsened after the 2017 media coverage (2·00 [0·84–3·15]). We did not observe effects in other minoritised ethnic groups.

**Interpretation:**

Our finding that the hostile environment policy worsened the mental health of people from Black Caribbean backgrounds in the UK suggests that sufficient, appropriate mental health and social welfare support should be provided to those affected. Impact assessments of new policies on minority mental health should be embedded in all policy making.

**Funding:**

Wellcome Trust.

## Introduction

Social inequalities are strongly associated with mental ill health.^1^ These inequalities can arise from a number of sources, including poverty,^2^ unemployment,^3^ housing insecurity,^4^ or restricted access to public services.^5^ There is evidence that these social determinants of mental ill health disproportionately affect people from minoritised ethnic backgrounds.^6^ Promoting the equitable social wellbeing of all citizens is a core function of government in a modern welfare state, such as the UK.^7^ Thus, conceptually, the effective implementation of government policies should also benefit population mental health, and be equitable within the population. However, when policies intentionally or unintentionally increase social inequalities, they act as structural determinants that can result in negative consequences for population mental health.

Over the past 10–15 years, migration, ethnic identity, and discrimination have received increased political, public, and media attention in light of several high profile international events, including the politicisation of immigration following a rise in populism globally; international and internal conflicts and the persecution of minoritised ethnic groups, resulting in the displacement of millions of people; and high-profile examples of systemic and institutional racism as exemplified by the murder of George Floyd in 2020. In the UK, fuelled by political rhetoric about immigration, the Government announced a series of immigration policy reforms from 2012 onwards, collectively known as the hostile environment policy.^8^ Several hundred Commonwealth citizens who had settled legally in the UK since World War 2 were falsely identified as undocumented and, in many cases, deported.^9^ This situation particularly affected people from Black Caribbean backgrounds who had immigrated to the UK since World War 2, known as the Windrush Generation.^10^ In addition to risk of deportation, the policy intended to make the UK a hostile environment for migrant and minoritised ethnic groups living in the UK.^8^, ^11^ The Immigration Act of 2014 required landlords, employers, the National Health Service, banks, and the police to check right-to-stay documentation.^12^ This requirement meant that many people from minoritised ethnic groups who did not have proof of citizenship lost jobs, incomes and benefits, housing, and access to public services.^10^ Furthermore, there have been reports that these checks have been addressed discriminatorily to people from minoritised backgrounds.^13^ Stories concerning the impact of the hostile environment policy, and the psychological distress it caused, became media headlines on Nov 28, 2017,^14^ and are collectively known as the Windrush scandal.^9^, ^10^

**Research in context**

**Evidence before this study**

In the past 10-15 years, migration, ethnic identity, and discrimination have received increased public and political attention, in light of anti-immigration policies, international conflicts, the persecution and displacement of minoritised ethnic groups, and high-profile examples of systemic and institutional racism. In 2012, the UK Government announced a series of immigration policy reforms, collectively known as the hostile environment policy. These reforms culminated in the Windrush scandal, whereby Commonwealth citizens who had settled legally in the UK before 1973, particularly those with Black Caribbean backgrounds, were falsely identified as undocumented and, in many cases, deported. Many people from minoritised ethnic backgrounds without proof of citizenship lost jobs, incomes and benefits, housing, and access to public services, and these policies increased structural racism and poverty. We performed three database searches for any relevant studies using the terms “hostile environment policy”; “Windrush”; and “immigration policy”, “mental health” and “UK” in PubMed, MEDLINE, Google Scholar, and Embase. To the best of our knowledge, ours is the first quantitative study to investigate the population-level effect of the hostile environment policy or the Windrush scandal on the mental health of minoritised ethnic groups.

**Added value of this study**

To the best of our knowledge, this is the first study to show that the hostile environment policy in the UK directly led to a decline in mental health for the people most exposed to these reforms. We showed that people of Black Caribbean backgrounds in the UK experienced worse mental health following the Immigration Act of 2014 (strongest for those born outside the UK), and following media coverage of the Windrush scandal in 2017 (strongest for those born in the UK).

**Implications of all the available evidence**

Our study builds on earlier qualitative studies and narrative reviews of the effect of immigration policies on mental health. Together, these findings suggest that political policies can produce, maintain, and exacerbate systemic inequities in population mental health. These findings demand that political systems and institutions prioritise development of accelerated reconciliation and primary prevention strategies that mitigate their social, moral, and public mental health injustices arising from more than a decade of discriminatory practices. Policy makers should ensure sufficient and appropriate clinical mental health resources are provided to meet any additional need that might arise from such policy decisions. It is essential that policy makers consider impact assessments on minority mental health before implementing new policies.

In this study, we investigated whether the effect of the hostile environment policy caused changes in the mental ill health of people from minoritised ethnic backgrounds in the UK. We specifically hypothesised that people from Black Caribbean backgrounds would have had worse mental ill health than people of White ethnicity following the introduction of the Immigration Act of 2014, and again after media coverage of the Windrush scandal commenced in 2017, because they were particularly targeted by the hostile environment policy and its aftermath, as encapsulated by the Windrush scandal.^9^, ^10^ We also investigated whether these effects were modified by migration status and household income. We hypothesised that the effect of these policies on mental ill health would be more pronounced among first-generation Black Caribbean migrants living in the UK than those who were UK-born, given the greater risks that migrants faced of being falsely identified as undocumented after the loss of immigration records in 2010. We also hypothesised that mental ill health following these policies would be worse for people from Black Caribbean backgrounds from lower household income quantiles, given that these individuals would have fewer resources to buffer the stressful effects of the hostile environment policy (such as threat of job loss, housing security, or access to legal advice).

## Methods

### Study design and participants

We performed a Bayesian interrupted time series analysis of longitudinal cohort data from the UK Household Longitudinal Study (UKHLS)^15^—a nationally representative panel study, with boost samples of key minoritised ethnic groups. We included all participants aged 16 years and older who had complete mental health data for our outcome of interest from at least one of 11 waves between 2009 until the first COVID-19 lockdown on March 23, 2020. We included participants from one of the exposed ethnic groups (Black Caribbean, Black African, Indian, Pakistani, and Bangladeshi) and the comparison ethnic group (White). We excluded participants who had missing data for sex and year of birth.

Special licence access to use the UKHLS data was approved by the data owner (project 233941). Ethical approval was received from the University College London Research Ethics Service (application 24211.001).

### Measures

We measured psychological distress using the 12-item General Health Questionnaire (GHQ-12; appendix pp 2–4), which is a widely used, well validated scale to screen for general (non-psychotic) mental health problems.^16^, ^17^ We treated GHQ-12 scores as a continuous outcome, with a minimum value of 0 (least psychological distress) and maximum value of 36 (most psychological distress).

We measured change in GHQ-12 scores across three exposure periods. Exposure period one consisted of interviews held during waves one to six, before the introduction of the 2014 Immigration Act (Aug 1, 2009–May 13, 2014). Exposure period two consisted of interviews held during waves four to nine, after the introduction of the 2014 Immigration Act and before the 2017 media coverage (May 14, 2014–Nov 27, 2017). Exposure period three consisted of interviews held during waves eight to 12, after mass media coverage began in 2017 and before the first COVID-19 lockdown in March, 2020 (Nov 28, 2017–March 23, 2020). We did not include data from any interviews held after the COVID-19 lockdowns began, as these were known to have had disproportionate adverse effects on mental health in minoritised ethnic groups.^18^ In each wave, participants were interviewed face to face, online, or by telephone at one point over a 24-month window (appendix p 5). This design meant that some survey waves could span two of our exposure periods. The exact interview date was recorded and used here to assign participants to the relevant exposure period.

We assessed whether there was a change in psychological distress after the policy implementation and after the media coverage by comparing mean GHQ-12 scores between each exposure period. We also assessed whether this change was persistent or transient by comparing mean GHQ-12 scores according to years since the start of each exposure period.

We compared changes in GHQ-12 scores across the three exposure periods, between participants of any White ethnicity (comparison group) and the ethnic minority groups considered to be potentially exposed to the hostile environment policies: Black Caribbean, Black African, Indian, Pakistani, and Bangladeshi. These are the most common minoritised ethnic groups in the UK according to the 2021 census; we excluded all other ethnic groups from the present study. We used self-report responses for ethnicity (survey participants were asked the question, “what is your ethnic group?”, with 18 possible response options identical to the ethnic categories used in the 2011 and 2021 Censuses of Great Britain; appendix p 6). We considered ethnicity to be a proxy measure for exposure to the hostile environment policy.

We included the following potential confounders, given they might affect both an individual's exposure to the effects of the Immigration Act 2014 and the 2017 media coverage, and an individual's mental ill health: sex, age, urban dwelling, relationship status, number of children, education level, health impairment, housing status, index of multiple deprivation, employment status, UK born, and total net household income. Detailed confounder descriptions are provided in the appendix (pp 7–9). We also investigated possible effect modification by migrant status (UK-born or first-generation), and total net household income (dichotomised at the national median).

### Statistical analysis

The UKHLS is weighted to account for the original sampling strategy and non-responses across waves. We created our own longitudinal weights based on the inverse probability of responses within each exposure period for individual participants and for each sampling stratum provided by UKHLS.^19^

We imputed missing covariate data using multiple imputation by chained equations, using the MICE package in R, with ten iterations. We pooled the imputed datasets, taking the mode of missing variables (all of which were categorical).^20^ All analyses were performed on the pooled dataset. Further information on this method is provided in the appendix (p 9). We reported the proportion of missingness for each variable included in the model, and differences in participant characteristics according to missing data.

We used a Bayesian interrupted time series linear model to estimate mean differences (MDs) and 95% credible intervals (CrIs) in GHQ-12 score across the three exposure periods in minoritised ethnic groups relative to the White ethnic group. An interrupted time series is a quasi-experimental design that can be used to evaluate the causal effects of an intervention on given outcomes over time, and it is being increasingly used to evaluate the effect of population-level policies.^21^ Further information on the interrupted time series design and statistical model is provided in the appendix (pp 7–9). Our model included ethnic group, the exposure period, all aforementioned confounders, a linear fixed effect for time (by year), a linear fixed effect for time since the start of each exposure period (by year), random effects to model residual temporal confounding (by year), and residual spatial confounding (by local authority area). These random effects accounted for variation not captured by our measured fixed effects. We specified weakly informative priors for all model parameters; these allow one to stabilise the inference while not imposing overbearing restrictions on the parameters’ values. We fitted the interrupted time series model using integrated nested Laplace approximations (INLAs) through the R-INLA package. INLA provides accurate approximations of the posterior distribution for all the model parameters, while avoiding the need for costly and time-consuming simulations such as Markov-chain Monte Carlo sampling.^22^

We then repeated this analysis, stratified first by whether participants were born in the UK, and second by net household income, to investigate effect moderation of these variables on the association between exposure to the hostile environment policy and subsequent media coverage and mental ill health. Finally, we repeated the primary analysis with three sensitivity analyses: first, without the use of weights to assess the effect of our weighting strategy; second, on participants with complete case data; and third, to assess the effect of non-response, by restricting our analysis to participants who responded at least once in each exposure period. We performed all analyses in R version 4.3.1.

We discussed this study, our findings, and our interpretation with two public representatives. The two public representatives also provided feedback on a draft of the study manuscript, which was included in the final submission.

### Role of the funding source

The funder of the study had no role in study design, data collection, data analysis, data interpretation, or writing of the report.

## Results

We included a total of 58 087 participants from the ethnicity groups of interest who had at least one response on the GHQ-12 part of the UKHLS during the study period. We excluded 7194 participants who did not respond to the GHQ-12 part of the UKHLS at least once during the study period (appendix pp 11–12). We excluded 2 participants with missing data for sex and year of birth. The mean age was 45·9 years (SD 34·6; range 16–106); 31 168 (53·6%) participants were female and 26 919 (46·3%) were male. Of the total sample, 78·90% were of White ethnicity, 4·34% were from Black African backgrounds, 3·78% were from Black Caribbean backgrounds, 5·43% were from Indian backgrounds, 4·82% were from Pakistani backgrounds, and 2·73% were from Bangladeshi backgrounds. We have reported the differences between minoritised ethnic groups compared with people of White ethnicity, and the proportion of missingness for all characteristics (table 1). The appendix (pp 11–16) reports differences between all UKHLS participants included in our study (58 087 [89·0%]) and those excluded due to totally missing GHQ-12 outcome data (7194 [11·0%]); differences between those included in our study with complete (87·1%) and missing data (12·9%) when at least one GHQ-12 score was reported; and differences between those who responded in every exposure period (36·4%) and those who did not (63·6%). We observed differences in the distribution of complete cases and those with some missing data by ethnicity, migrant status, age, urban residency, housing status, income, and education and deprivation level, but not by sex, marital status, number of children, or health impairment status.

**Table 1:** Characteristics of individuals included in the analysis, measured at the date the Immigration Act of 2014 was implemented

		**Total sample**	**White ethnicity (controls)**	**Black African ethnicity**	**Black Caribbean ethnicity**	**Indian ethnicity**	**Pakistani ethnicity**	**Bangladeshi ethnicity**
Total		58 087 (100·0%)	45 833 (78·9%)	2519 (4·3%)	2197 (3·8%)	3153 (5·4%)	2801 (4·8%)	1584 (2·7%)
Age in years*		44 (29–60)	47 (31–63)	35 (24–46)	43 (26–55)	38 (27–52)	33 (23–44)	32 (22–42)
	Kruskal-Wallis test	..	Ref	p<0·0001	p<0·0001	p<0·0001	p<0·0001	p<0·0001
Female		31 168 (53·7%)	24 649 (53·8%)	1411 (56·0%)	1299 (59·1%)	1542 (48·9%)	1467 (52·4%)	800 (50·5%)
Male		26 919 (46·3%)	21 184 (46·2%)	1108 (44·0%)	898 (40·9%)	1611 (51·1%)	1334 (47·6%)	784 (49·5%)
	χ^2^ test	..	Ref	p=0·0285	p<0·0001	p<0·0001	p=0·1475	p=0·0102
UK born		41 047 (70·7%)	36 099 (78·8%)	527 (20·9%)	1351 (61·5%)	1147 (36·4%)	1234 (44·1%)	689 (43·5%)
	Missing	5898 (10·2%)	5658 (12·3%)	36 (1·4%)	19 (0·9%)	82 (2·6%)	56 (2·0%)	36 (2·3%)
	χ^2^ test	..	Ref	p<0·0001	p<0·0001	p<0·0001	p<0·0001	p<0·0001
Marital status								
	In relationship	43 387 (74·7%)	32 695 (71·3%)	2100 (83·4%)	1669 (76·0%)	2862 (90·8%)	2585 (92·3%)	1476 (93·2%)
	Missing	52 (0·0%)	29 (0·0%)	7 (0·3%)	6 (0·3%)	5 (0·2%)	1 (0·0%)	4 (0·3%)
	χ^2^ test	..	Ref	p<0·0001	p<0·0001	p<0·0001	p<0·0001	p<0·0001
Number of children*		0 (0–1)	0 (0–0)	0 (0–2)	0 (0–0)	0 (0–1)	0 (0–2)	0 (0–2)
	Missing	0	0	0	0	0	0	0
	Kruskal-Wallis test	..	Ref	p<0·0001	p=0·2597	p<0·0001	p<0·0001	p<0·0001
Any health impairment		18 280 (31·5%)	15 813 (34·5%)	343 (13·6%)	672 (30·6%)	592 (18·8%)	576 (20·7%)	284 (17·9%)
	Missing	15 (0·0%)	7 (0·0%)	0	2 (0·0%)	4 (0·1%)	0 (0·0%)	2 (0·1%)
	χ^2^ test	−	Ref	p<0·0001	p<0·0001	p<0·0001	p<0·0001	p<0·0001
Urban dwelling		47 906 (82·5%)	35 762 (78·0%)	2492 (98·9%)	2168 (98·7%%)	3110 (98·6%)	2796 (99·8%)	1578 (99·6%)
	Missing	0	0	0	0	0	0	0
	χ^2^ test	..	Ref	p<0·0001	p<0·0001	p<0·0001	p<0·0001	p<0·0001
Housing status								
	Owned	37 364 (64·3%)	31 020 (67·7%)	569 (22·6%)	965 (43·9%)	2275 (72·2%)	1937 (69·2%)	598 (37·8%)
	Private rented	10 758 (18·5%)	7275 (15·9%)	1255 (49·8%)	907 (41·3%)	225 (7·1%)	378 (13·5%)	718 (45·3%)
	State rented	9701 (16·7%)	7371 (16·1%)	668 (26·5%)	315 (14·3%)	629 (20·0%)	461 (16·5%)	257 (16·2%)
	Missing	264 (0·5%)	167 (0·4%)	27 (1·1%)	10 (0·5%)	24 (0·8%)	25 (0·9%)	11 (0·7%)
	χ^2^ test	..	Ref	p<0·0001	p<0·0001	p<0·0001	p<0·0001	p<0·0001
Highest education								
	Postgraduate	6052 (10·4%)	4 889 (10·7%)	331 (13·1%)	290 (13·2%)	260 (8·3%)	194 (6·9%)	88 (5·6%)
	Undergraduate	12 827 (22·1%)	9675 (21·1%)	757 (30·1%)	407 (18·5%)	1095 (34·7%)	569 (20·3%)	324 (21·3%)
	A level	12 279 (21·1%)	9802 (21·4%)	528 (21·0%)	497 (22·6%)	558 (17·7%)	531 (19·0%)	363 (16·9%)
	GCSE	12 820 (22·1%)	10 355 (22·6%)	431 (17·1%)	521 (23·7%)	512 (16·2%)	612 (21·9%)	389 (18·1%)
	Other	5263 (9·1%)	4435 (9·7%)	164 (6·5%)	192 (8·7%)	186 (5·9%)	194 (6·9%)	92 (6·0%)
	None	7514 (12·9%)	6082 (13·3%)	166 (6·6%)	238 (10·8%)	313 (9·9%)	447 (16·0%)	268 (17·6%)
	Missing	1332 (2·3%)	595 (1·3%)	142 (5·6%)	52 (2·4%)	229 (7·3%)	254 (9·1%)	60 (3·9%)
	χ^2^ test	..	Ref	p<0·0001	p<0·0001	p<0·0001	p<0·0001	p<0·0001
Employment status								
	Employed	26 177 (45·1%)	21 072 (46·0%)	1133 (45·0%)	968 (44·1%)	1562 (49·5%)	872 (31·1%)	570 (37·4%)
	Self-employed	4384 (7·6%)	3541 (7·7%)	135 (5·4%)	142 (6·5%)	247 (7·8%)	232 (8·3%)	87 (5·7%)
	Unemployed	5632 (9·7%)	4004 (8·7%)	321 (12·7%)	399 (18·2%)	282 (8·9%)	406 (14·5%)	220 (14·4%)
	Retired	11 495 (19·8%)	10 610 (23·2%)	106 (4·2%)	285 (13·0%)	306 (9·7%)	135 (4·8%)	53 (3·5%)
	Family leave	3720 (6·4%)	2267 (5·0%)	208 (8·3%)	91 (4·1%)	284 (9·0%)	586 (20·9%)	284 (18·6%)
	Student	6668 (11·5%)	4336 (9·5%)	615 (1·3%)	310 (14·1%)	470 (14·9%)	568 (20·3%)	369 (24·2%)
	Missing	11 (0·0%)	3 (0·0%)	1 (0·0%)	2 (0·00%)	2 (0·0%)	2 (0·0%)	1 (0·0%)
	χ^2^ test	..	Ref	p<0·0001	p<0·0001	p<0·0001	p<0·0001	p<0·0001
Household monthly income								
	6 (highest)	6545 (11·3%)	4490 (9·8%)	382 (15·2%)	298 (13·6%)	437 (13·9%)	588 (21·0%)	350 (22·1%)
	5	4938 (8·5%)	3983 (8·6%)	219 (8·7%)	158 (7·2%)	276 (8·8%)	203 (7·3%)	99 (6·3%)
	4	6826 (11·8%)	5864 (12·8%)	209 (8·3%)	197 (9·0%)	355 (11·3%)	126 (4·5%)	75 (4·7%)
	3	10 393 (17·9%)	8778 (19·2%)	307 (12·2%)	339 (15·4%)	571 (18·1%)	241 (8·6%)	157 (9·9%)
	2	18 100 (31·2%)	14 516 (31·7%)	746 (29·6%)	712 (32·4%)	900 (28·5%)	788 (28·1%)	438 (27·7%)
	1 (lowest)	11 121 (19·2%)	8133 (17·7%)	618 (24·5%)	480 (21·9%)	593 (18·8%)	840 (30·0%)	457 (28·9%)
	Missing	164 (0·3%)	69 (0·2%)	38 (1·5%)	13 (0·6%)	21 (0·7%)	15 (0·5%)	8 (0·5%)
	χ^2^ test	..	Ref	p<0·0001	p<0·0001	p<0·0001	p<0·0001	p<0·0001
Index of multiple deprivation decile								
	10 (highest)	4937 (8·5%)	4710 (10·3%)	23 (0·9%)	44 (2·0%)	132 (4·2%)	19 (0·7%)	9 (0·6%)
	9	5159 (8·9%)	4861 (10·6%)	56 (2·2%)	55 (2·5%)	156 (5·0%)	25 (0·9%)	6 (0·4%)
	8	5325 (9·9%)	4894 (10·7%)	85 (3·4%)	52 (2·4%)	228 (7·2%)	56 (2·0%)	10 (0·6%)
	7	5535 (9·5%)	5003 (10·9%)	94 (3·7%)	89 (4·1%)	227 (7·2%)	94 (3·4%)	28 (1·8%)
	6	5556 (9·6%)	4846 (10·6%)	132 (5·2%)	165 (7·5%)	261 (8·3%)	102 (3·6%)	50 (3·2%)
	5	5613 (9·7%)	4652 (10·2%)	166 (6·6%)	201 (9·2%)	344 (10·9%)	160 (5·7%)	90 (5·7%)
	4	5634 (9·7%)	4265 (9·3%)	284 (11·3%)	286 (13·0%)	426 (13·5%)	197 (7·0%)	176 (11·1%)
	3	6644 (11·4%)	4276 (9·3%)	578 (23·1%)	417 (19·0%)	589 (18·7%)	396 (14·1%)	388 (24·5%)
	2	7034 (12·1%)	4183 (9·1%)	635 (25·2%)	494 (22·5%)	520 (16·5%)	648 (23·2%)	554 (35·0%)
	1 (lowest)	6650 (11·5%)	4143 (9·0%)	466 (8·5%)	394 (17·9%)	270 (8·6%)	1104 (39·4%)	273 (17·2%)
	Missing	0	0	0	0	0	0	0
	χ^2^ test	..	Ref	p<0·0001	p<0·0001	p<0·0001	p<0·0001	p<0·0001
Responses		4 (2–9)	5 (2–9)	2 (1–5)	3 (1–6)	3 (1–6)	3 (1–5)	2 (1–5)
	Kruskal-Wallis test	..	Ref	p<0·0001	p<0·0001	p<0·0001	p<0·0001	p<0·0001
Present in each exposure period		21 129 (36·4%)	18 600 (40·6%)	397 (15·8%)	504 (22·9%)	748 (23·7%)	561 (20·0%)	319 (20·1%)
	χ^2^ test	..	Ref	p<0·0001	p<0·0001	p<0·0001	p<0·0001	p<0·0001
Complete covariate data		50 585 (87·1%)	39 470 (86·1%)	2290 (90·9%)	2073 (94·4%)	2803 (88·9%)	2463 (87·9%)	1486 (93·8%)
	χ^2^ test	..	Ref	p<0·0001	p<0·0001	p<0·0001	p=0·0068	p<0·0001
GHQ-12 score before Immigration Act 2014		11·1 (5·5)	11·1 (5·4)	10·3 (5·8)	11·4 (6·0)	10·9 (5·8)	11·9 (6·3)	11·7 (6·0)
	*t* test	..	Ref	p<0·0001	p=0·0114	p=0·0453	p<0·0001	p<0·0001

**Da**ta shown are n (%) or median (IQR). Kruskal-Wallis tests, χ^2^ tests, and *t* tests compared characteristics between minoritised ethnic groups and the White ethnic group. GHQ-12-12-item General Health Questionnaire. GCSE=General Certificate of Secondary Education.

* At the time of the 2014 Immigration Act.

The mean GHQ-12 score before the Immigration Act 2014 in people from White ethnic backgrounds was 11·1 (SD 5·4) out of a possible maximum of 36. We detected small differences in pre-Immigration Act GHQ-12 scores between ethnic groups (table 1), which varied from lowest psychological distress in those of Black African heritage to highest distress in those of Pakistani heritage.

In our main analysis (table 2), we found evidence of greater psychological distress in people from Black Caribbean backgrounds than White participants after implementation of the Immigration Act 2014 (MD 0·67, 95% CrI 0·06 to 1·28). This effect persisted for several years, shown by the absence of difference over time since implementation of the Immigration Act 2014. We also found evidence that the Black Caribbean group had a further increase in psychological distress relative to White participants after the Windrush scandal media coverage commenced in 2017 (MD 1·28, 95% CrI 0·34 to 2·21; table 2). This effect did not diminish over time since the media coverage (MD –0·07, 95% CrI –0·51 to 0·37). We found no change in psychological distress for any other minoritised ethnic group compared with people from the White ethnic group in either period (figure).

**Table 2:** Differences in mean GHQ-12 scores across each exposure period, and according to the number of years since the start of each exposure period, between different ethnic groups following interrupted time series analysis

	**Exposure period 2 (post Immigration Act 2014)**	**Time since start of exposure period 2, years**	**Exposure period 3 (post media coverage 2017)**	**Time since start of exposure period 3, years**
Black African (n=2519)	0·53 (−0·12 to 1·17)	−0·32 (−0·62 to −0·01)*	0·54 (−0·44 to 1·52)	0·45 (0·00 to 0·91)
Black Caribbean (n=2197)	0·67 (0·06 to 1·28)	−0·12 (−0·40 to 0·17)	1·28 (0·34 to 2·21)	−0·07 (−0·51 to 0·37)
Indian (n=3153)	0·35 (−0·20 to 0·89)	−0·19 (−0·44 to 0·06)	0·08 (−0·69 to 0·85)	0·28 (−0·08 to 0·63)
Pakistani (n=2801)	0·27 (−0·33 to 0·88)	−0·53 (−0·81 to −0·25)*	0·52 (−0·32 to 1·37)	−0·25 (−0·13 to 0·62)
Bangladeshi (n=1584)	−0·43 (−1·24 to 0·38)	0·20 (−0·57 to 0·17)	−0·66 (−1·85 to 0·53)	0·19 (−0·36 to 0·73)
White (n=45 833)	Ref	Ref	Ref	Ref

Data shown are mean difference (credible interval). GHQ-12=General Health Questionnaire 12-item version.

* In people from Black African and Pakistani ethnic backgrounds, we saw no evidence of a change after the implementation of the Immigration Act of 2014. However, we do see a gradual improvement in mental health (reduction in GHQ-12 score) in these individuals in the years following the Immigration Act of 2014 (this is not interpretable as a causal effect of the Immigration Act of 2014).

**Figure:**
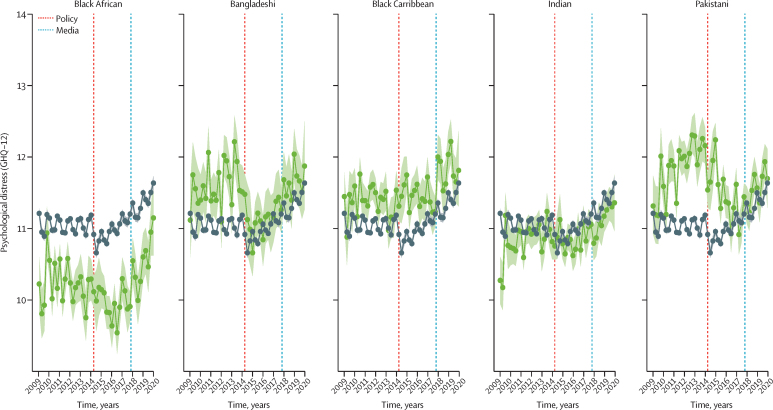
Effects of the hostile environment policy on mental ill health across different ethnic groups compared with people of White ethnicity Green data points and lines represent mean GHQ-12 scores from the respective ethnic minority groups; black data points and lines represent mean GHQ-12 scores from the White ethnicity group. The red dashed line shows the implementation of the Immigration Act 2014 and the blue dashed line shows the start of the Windrush scandal media coverage. GHQ-12=General Health Questionnaire 12-item version.

Following the introduction of the Immigration Act 2014, we observed an increase in psychological distress in first-generation migrants from Black Caribbean backgrounds relative to the White group (MD 1·25, 95% CrI 0·11–2·38; table 3). We observed no change for UK-born Black Caribbean participants in this period, but their psychological distress increased relative to the White group after media coverage began (2·00, 0·84–3·15). We observed no changes in psychological distress by migrant status for any other minoritised ethnic group, except for an increase in psychological distress following the introduction of the Immigration Act 2014 in UK-born Black African participants (1·39, 0·04–2·73; table 3).

**Table 3:** Differences in mean GHQ-12 scores across each exposure period, and according to the number of years since the start of each exposure period, by ethnicity and migrant status following interrupted time series analysis

	**Exposure period 2 (post Immigration Act 2014)**	**Time since start of exposure period 2, years**	**Exposure period 3 (post media coverage 2017)**	**Time since start of exposure period 3, years**
**UK-born groups**				
Black African (n=527)	1·39 (0·04 to 2·73)	−0·40 (−1·04 to 0·25)	1·22 (−0·80 to 3·23)	0·64 (−0·30 to 1·57)
Black Caribbean (n=1351)	0·52 (−0·24 to 1·29)	−0·17 (−0·53 to 0·19)	2·00 (0·84 to 3·15)	−0·27 (−0·81 to 0·27)
Indian (n=1147)	0·68 (−0·22 to 1·58)	−0·23 (−0·65 to 0·18)	0·60 (−0·66 to 1·86)	0·24 (−0·33 to 0·81)
Pakistani (n=1234)	−0·19 (−1·08 to 0·69)	−0·48 (−0·89 to −0·07)	0·26 (−0·96 to 1·48)	0·20 (−0·34 to 0·74)
Bangladeshi (n=689)	−1·44 (−2·62 to −0·26)	−0·00 (−0·55 to 0·55)	−1·27 (−2·99 to 0·45)	0·04 (−0·75 to 0·82)
White (n=36 099)	Ref	Ref	Ref	Ref
**Non-UK-born groups**				
Black African (n=1956)	0·27 (−0·59 to 1·13)	−0·21 (−0·61 to 0·21)	0·25 (−1·06 to 1·55)	0·29 (−0·32 to 0·89)
Black Caribbean (n=799)	1·25 (0·11 to 2·38)	−0·32 (−0·85 to 0·19)	0·34 (−1·42 to 2·09)	0·42 (−0·40 to 1·25)
Indian (n=1924)	0·46 (−0·36 to 1·27)	−0·35 (−0·73 to 0·20)	0·09 (−1·10 to 1·27)	0·36 (−0·19 to 0·90)
Pakistani (n=1511)	0·86 (−0·08 to 1·81)	−0·76 (−1·20 to −0·32)	0·67 (−0·68 to 2·01)	0·44 (−0·17 to 1·04)
Bangladeshi (n=876)	0·48 (−0·70 to 1·66)	−0·46 (−1·00 to 0·08)	0·16 (−1·63 to 1·94)	0·33 (−0·48 to 1·14)
White (n=4076)	Ref	Ref	Ref	Ref

Data shown are mean difference (credible interval). GHQ-12=General Health Questionnaire 12-item version.

There was no evidence of effect modification by income level on changes in psychological distress following either the Immigration Act 2014 or the 2017 media coverage (table 4). For example, compared with White participants, effect sizes indicative of worse mental health were similar for higher and lower income Black Caribbean groups following both the Immigration Act 2014 and 2017 media coverage (table 4). However, uncertainty around these effects was generally high and included the line of null effect, except for greater psychological distress in lower income Black Caribbean participants after media coverage began.

**Table 4:** Differences in mean GHQ-12 scores across each exposure period, and according to the number of years since the start of each exposure period, by ethnicity and net household income following interrupted time series analysis

	**Exposure period 2 (post policy implementation)**	**Time since start of exposure period 2, years**	**Exposure period 3 (post media coverage)**	**Time since start of exposure period 3, years**
**High household income group**				
Black African (n=810)	0·41 (−0·71 to 1·54)	−0·09 (−0·63 to 0·44)	1·59 (−0·14 to 3·33)	−0·24 (−1·07 to 0·60)
Black Caribbean (n=653)	0·66 (−0·42 to 1·74)	− 0·28 (−0·80 to 0·44)	1·20 (−0·47 to 1·74)	0·04 (−0·76 to 0·84)
Indian (n=1068)	0·23 (−0·67 to 1·12)	−0·25 (−0·63 to 0·16)	0·20 (−1·09 to 1·50)	0·12 (−0·48 to 0·72)
Pakistani (n=917)	0·98 (−0·16 to 2·13)	−1·13 (−1·64 to −0·61)	0·93 (−0·67 to 2·53)	0·12 (−0·48 to 0·72)
Bangladeshi (n=524)	1·72 (−0·74 to 2·19)	−0·86 (−1·54 to −0·19)	1·09 (−0·13 to 3·30)	0·87 (0·15 to 1·59)
White (n=14 337)	Ref	Ref	Ref	Ref
**Low household income group**				
Black African (n=1671)	0·40 (−0·41 to 1·17)	−0·33 (−0·70 to 0·04)	−0·05 (−1·24 to 1·14)	0·62 (0·08 to 1·16)
Black Caribbean (n=1531)	0·62 (−0·12 to 1·36)	−0·12 (−0·47 to 0·22)	1·37 (0·24 to 2·50)*	−0·13 (−0·65 to 0·40)
Indian (n=2064)	0·63 (−0·05 to 1·31)	−0·31 (−0·62 to 0·00)	0·40 (−0·57 to 1·36)	0·43 (−0·01 to 0·87)
Pakistani (n=1869)	0·07 (−0·64 to 0·78)	−0·34 (−0·67 to −0·01)	0·47 (−0·52 to 1·46)	0·03 (−0·41 to 0·47)
Bangladeshi (n=1052)	−0·80 (−1·76 to 0·16)	0·02 (−0·42 to 0·46)	−0·97 (−2·39 to 0·45)	0·15 (−0·50 to 0·80)
White (n=31 427)	Ref	Ref	Ref	Ref

Data shown are mean difference (credible interval). GHQ-12=General Health Questionnaire 12-item version.

Our results remained similar throughout our three sensitivity analyses performed without weighting, using complete cases only, and restricted to participants who had responded at least once in each exposure period (appendix pp 17–19), albeit with lower precision when restricted to participants who had responded at least once in each exposure period due to the substantially reduced sample size (n=21 129).

## Discussion

Our study shows that political policies can produce, maintain, and exacerbate systemic inequities in population mental health. We found evidence that the UK Government's hostile environment policy and subsequent media coverage of the Windrush scandal caused people of Black Caribbean ethnicities living in the UK to experience greater psychological distress relative to the White ethnicity group, in line with our hypothesis. This mental health inequality persisted for several years after these events. In first-generation Black Caribbean migrants, higher levels of psychological distress occurred immediately after the introduction of the Immigration Act 2014, but those did not rise further when the Windrush scandal emerged in the popular press. In UK-born people of Black Caribbean heritage, greater psychological distress occurred in the aftermath of the 2017 Windrush scandal media coverage, and this was the largest single change in mental health observed in our study.

We acknowledge some important limitations of our study. First, although we used the GHQ-12, a commonly used, cross-culturally validated instrument to assess psychological distress, we recognise that a single instrument might not capture the full range of distress that people exposed to the hostile environment policy will have experienced, including, for example, moral injury,^23^ psychosocial disempowerment,^24^ or failures of justice.^25^ Delineating the total impact of this policy on harm to human health and wellbeing is an important area for further research, including qualitative studies that seek to deepen our understanding of lived experience narratives, and the mechanisms through which distress can arise. Such research might also illuminate why we did not observe changes in psychological distress for other minoritised ethnic groups in this study. Second, we were unable to assess the effect of policies on prevalences of mental disorders that meet clinical criteria for diagnosis. Third, we might have had insufficient power to detect the impact of the hostile environment policy on mental health outcomes in some subgroup analyses. For example, we observed greater statistical uncertainty in our results stratified by ethnicity and income compared with the main analysis (stratified by ethnicity alone), potentially driven by smaller sample sizes in these analyses, although for the Black Caribbean group (and others), no apparent effect modification by income was observed. For this reason, we did not examine differential effects according to other participant characteristics, such as sex. Finally, we used ethnic group as a proxy for exposure to the hostile environment policy because we were unable to determine the extent to which people were directly exposed to the policy. For example, we did not have information on whether people were undocumented, or whether they had directly experienced stressors arising from the policy, such as legal action, job loss, or housing insecurity.

Since 2017, there have been numerous media reports on the impact of the hostile environment policy on people's livelihoods and mental wellbeing.^9^, ^10^ Our quasi-experimental study provides some of the first evidence that this policy and its aftermath had a causal effect on increased psychological distress in the Black Caribbean population in the UK. This injustice will exacerbate health inequalities known to already exist for this group, who, like several other minoritised ethnic groups, face greater risk of being diagnosed with serious mental illnesses,^26^ and who face systemic and institutionalised racism that result in gross injustices in pathways to care^27^ and treatment within mental health services.^28^

Our findings also show the intersectional ways in which ethnicity, migrant status, and income level combine as structural forces that erode the agency required for affected individuals to achieve equitable levels of mental health to the general population. As such, our results provide crucial insights for policy makers globally who are responsible for designing and implementing any policy with the potential to impinge on social justice and public health equity. Our results demand that our political systems and institutions prioritise development of accelerated reconciliation and primary prevention strategies that mitigate their social, moral, and public (mental) health injustices arising from more than a decade of discriminatory practices. Although the UK Home Office reports issuing apologies to 51 individuals affected by deportation,^29^ this is not sufficient to rectify the broader social, economic, and mental health harms sustained at a population level. In addition to deportation of some individuals, the hostile environment policy resulted in increased structural and institutional racism, whereby affected individuals lost jobs, incomes and benefits, housing, and access to public services.^10^ There is strong evidence that these sources of social disadvantage are associated with mental ill health.^1^ Our finding that these policies led to worse mental health of UK-born Black Caribbean participants following media coverage of the Windrush scandal is consistent with evidence from previous research that media exposure to distressing news concerning people from the same minoritised ethnic background is associated with mental ill health.^30^ Policy makers should ensure sufficient and appropriate clinical mental health resources are provided to meet any additional need that might arise from such policy decisions; however, this will be particularly challenging when the same structural forces affecting these needs also generate mistrust of the mental health system that prevents timely access to care. Ultimately, governments should ensure that future policies are not implemented without consideration of the effects they could have on public mental health, via a population mental health impact assessment that quantifies the potential effect (negative or positive) that any policy could have on overall levels of public mental health or on exacerbating or reducing inequalities by ethnicity or any other protected characteristic.

## Data sharing

Data are available via the UK Household Longitudinal Study (https://www.understandingsociety.ac.uk/).

## Declaration of interests

We declare no competing interests.
