# Supplementary material for: The effect of immigration policy reform on mental health in people from minoritised ethnic groups in England: an interrupted time series analysis of longitudinal data from the UK Household Longitudinal Study cohort
Source: Lancet Psychiatry. 2024 Mar;11(3):183–92. doi: 10.1016/S2215-0366(23)00412-1 (PMC11101585; doi:10.1016/S2215-0366(23)00412-1)
Supplement: Supplementary appendix [file mmc1.pdf]

THE LANCET
Psychiatry

Supplementary appendix

This appendix formed part of the original submission and has been peer reviewed. We post it as supplied by the authors.

Supplement to: Jeffery A, Gascoigne C, Dykxhoorn J, et al. The effect of immigration policy reform on mental health in people from minoritised ethnic groups in England: an interrupted time series analysis of longitudinal data from the UK Household Longitudinal Study cohort. *Lancet Psychiatry* 2024; **11:** 183–92.

SUPPLEMENTARY MATERIAL

Table of contents

Supplementary File A – General Health Questionnaire (GHQ) – 12 Page 2

Supplementary File B – Study design timeline Page 5

Supplementary File C – Ethnicity survey question with responses Page 6

Supplementary File D – Bayesian Interrupted Time Series design, model specification & multiple imputation by chained equations Page 7

Supplementary File E – Missing Data Page 11

Supplementary File F – Sensitivity Analyses Page 17

Supplementary File A – General Health Questionnaire (GHQ) – 12

The next questions are about how you have been feeling over the last few weeks.

Have you recently been able to concentrate on whatever you're doing?

Option	Label
1	Better than usual
2	Same as usual
3	Less than usual
4	Much less than usual

Have you recently lost much sleep over worry?

Option	Label
1	Not at all
2	No more than usual
3	Rather more than usual
4	Much more than usual

Have you recently felt that you were playing a useful part in things?

Option	Label
1	More so than usual
2	Same as usual
3	Less so than usual
4	Much less than usual

Have you recently felt capable of making decisions about things?

Option	Label
1	More so than usual
2	Same as usual
3	Less so than usual
4	Much less capable

Have you recently felt constantly under strain?

Option	Label
1	Not at all
2	No more than usual
3	Rather more than usual

4 Much more than usual

Have you recently felt you couldn't overcome your difficulties?

Option	Label
3	Rather more than usual
1	Not at all
2	No more than usual
4	Much more than usual

Have you recently been able to enjoy your normal day-to-day activities?

Option	Label
1	More so than usual
2	Same as usual
3	Less so than usual
4	Much less than usual

Have you recently been able to face up to problems?

Option	Label
1	More so than usual
2	Same as usual
3	Less able than usual
4	Much less able

Have you recently been feeling unhappy or depressed?

Option	Label
1	Not at all
2	No more than usual
3	Rather more than usual
4	Much more than usual

Have you recently been losing confidence in yourself?

1	Not at all
2	No more than usual
3	Rather more than usual
4	Much more than usual

Have you recently been thinking of yourself as a worthless person?

Option	Label
1	Not at all
2	No more than usual
3	Rather more than usual
4	Much more than usual

Have you recently been feeling reasonably happy, all things considered?

Option	Label
1	More so than usual
2	About the same as usual
3	Less so than usual
4	Much less than usual

Scoring: Option 1 = 0 points; Option 2 = 1 point; Option 3 = 2 points; Option 4 = 3 points. Minimum of 0 points indicates no psychological distress. Maximum value of 36 points indicates maximum psychological distress.

Supplementary File B – Study design timeline

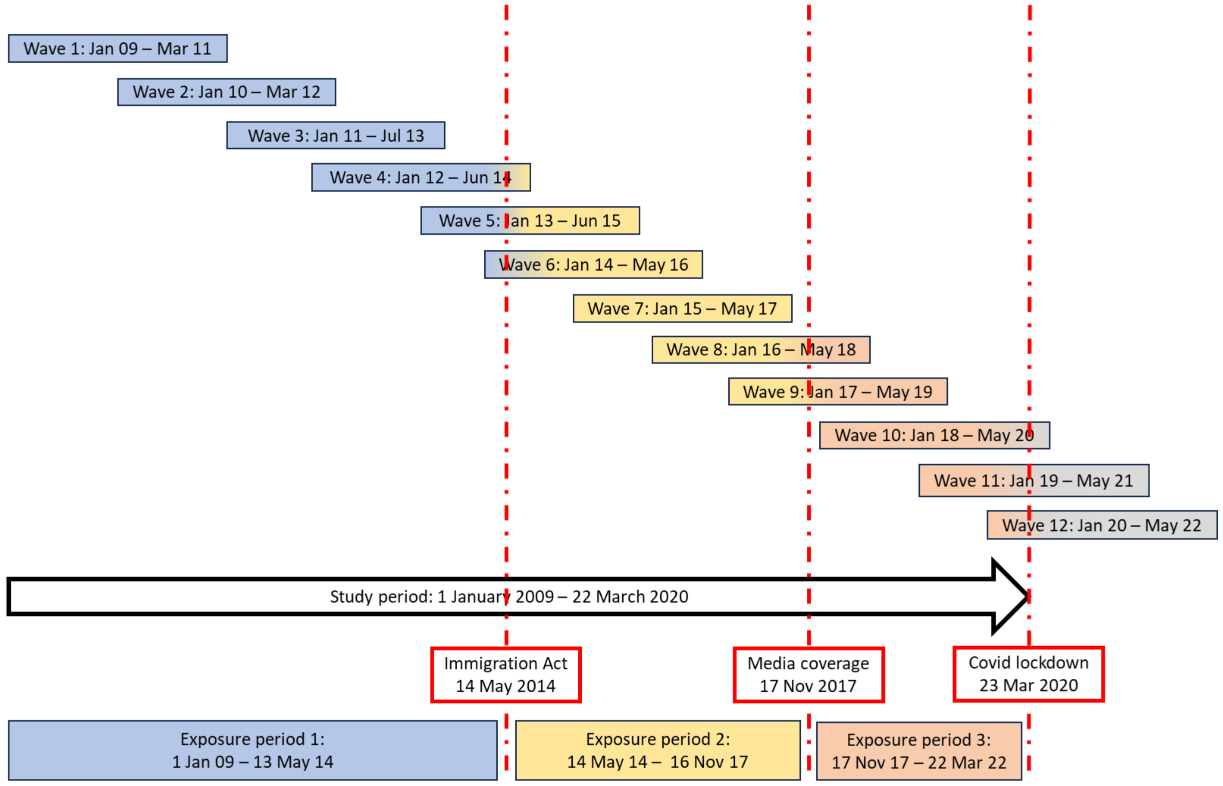

Supplementary File C – Ethnicity survey question with responses

Text: What is your ethnic group?

Option	Label
1	British
2	Irish
3	Gypsy/Irish Traveller
4	Other White background
5	White & Black Caribbean
6	White and Black African
7	White and Asian
8	Other mixed background
9	Indian
10	Pakistani
11	Bangladeshi
12	Chinese
13	Other Asian background
14	Caribbean
15	African
16	Other Black background
17	Arab
97	Any other ethnic group

Supplementary File D – Bayesian Interrupted Time Series design, model specification & multiple imputation by chained equations

Bayesian Interrupted Time Series

The ITS design uses a time series of the outcome to establish an underlying trend, which is 'interrupted' by the policy at a known point in time. The effect of this intervention can be evaluated as the change in the outcome before and after the intervention in the exposed group (here, minoritised ethnic groups) relative to the change in the outcome before and after the intervention in the comparison group (here, the white group). This quasi-experimental design has a causal interpretation because it emulates a randomised-controlled trial: if we assume that participants within each group immediately before and after the intervention are exchangeable, then we can emulate a situation in which the intervention (i.e. the policy) was randomly allocated to those before and after (that is, that confounders, observed or unobserved, would be balanced before and after the introduction of the intervention). Further, the ITS design implicitly isolates the effect of the intervention from other potentially co-occurring events (such as Brexit), provided that such events affected the exposed and comparison groups to the same extent (C1).

Model specification

We fitted our ITS model as follows:

$$\begin{aligned} y = intercept & \\ & + [(exposure\ period) + (time) + (time\ since\ exposure\ period\ 2) \\ & + (time\ since\ exposure\ period\ 3)]\ X\ (ethnicity\ group) \\ & + (fixed\ effects\ for\ confounders) \\ & + (random\ spatial\ and\ temporal\ effects) \end{aligned}$$

Exposure period: This was categorised as 1, 2, and 3. Where 1 = all interviews dated before the implementation of the Immigration Act of 2014; 2 = all interviews dated after the implementation Act of 2014, but before the start of the Windrush media coverage in 2017; 3 = all interviews dated after the start of the Windrush media coverage in 2017.

Time: Time was defined by a numerical variable representing the year in which the interview took place.

Time since exposure period 2: This was defined as the time in years since the implementation of the Immigration Act of 2014. Years 2014 and previous were defined as zero. Subsequent years were defined as 1-6, starting in 2015 and ending in 2020.

Time since exposure period 3: This was defined as the time in years since the start of the Windrush media coverage in 2017. Years 2017 and previous were defined as zero. Subsequent years were defined as 1-3, starting in 2018 and ending in 2020.

Confounders:

- Sex (dichotomised as male, female)
- Age in years
- Urban dwelling (dichotomised as urban – area with population of 10,000 or more; rural – area with population less than 10,000)
- Relationship status (dichotomised as in relationship, not in relationship)
- Number of children (continuous count)
- Highest educational qualification (categorised as higher degree, undergraduate degree, A-Level, GCSE, other, none)
- Physical or mental health impairment or disability lasting more than 12 months (dichotomised as yes, no),
- Housing status (categorised as owned, privately rented, non-privately rented)
- Index of multiple deprivation (IMD) decile (the official measure of relative deprivation in England, based on 39 indicators in 7 domains – income, employment, health deprivation and disability, education and skills training, crime, barriers to housing and services, living environment. We used the 2019 IMD rank based for the local authority area that the participant lived in (C2),
- Employment status categorised as employed, unemployed or long-term sick/disabled, retired, student)
- UK born (dichotomised as yes, no)
- Total net household income (categorised according to annual national quintiles).
- Time in years as a continuous variable

Sex and country of birth were measured in the first interview a participant responded to. The remaining confounders were treated as time-varying. If a participant did not have a response for any time-varying confounder in any given year, their response for that year was filled from their actual response in the closest year. If a participant never responded to a specific confounder, this was filled using multiple imputation.

Temporal structured random effect: We used a Random Walk of order two (C3) model to capture local correlations in time between the given year and the two years before and after. This specification is very flexible and approximates highly non-linear time trends, while keeping computational complexity at a manageable level. This random effect accounted for any residual temporal variation in psychological distress. This could be due to natural changes in the prevalence of mental ill health or due to other events that could influence mental ill health.

Spatial structured and unstructured random effect: We used a Besag-York-Mollie Two (BYM2) (C4-5) model, which is commonly used for disease mapping to capture both global and local correlations between neighbouring local authorities. The global correlations capture similarities between Local Authorities regardless of whether they are neighbours or not, and the local correlations capture the similarities in the response between neighbouring Local Authorities.

Multiple Imputation by Chained Equations

We imputed missing covariate data using multiple imputation by chained equations (MICE), using the MICE package in R, with 10 iterations (20). Our imputation model included all variables used for analysis in the dataset. We pooled the imputed datasets, taking mode imputed variables (all of which were categorical) (C6). All analyses were performed on a single pooled dataset. We chose this method of imputation and analysis to limit the computational burden – pooling together a large number of imputed datasets, each with the relevant simulations from the posterior distribution would have added a non-trivial computational burden, given the size of the dataset and the rich model structure we have selected. Our pragmatic approach preserved the statistical uncertainty between participants, but slightly under-characterise the between-imputation uncertainty in the data. Nonetheless, analyses restricted to our complete case sample (Supplementary File E, Table DE), as well as small-scale tests on a smaller number of imputed datasets, gave similar results to our imputed results, suggesting our method did not substantially bias our estimates.

References

C1. Kim Y, Steiner P. *Quasi-Experimental Designs for Causal Inference.* Educ Psychol. 2016;51(3-4):395-405. doi: 10.1080/00461520.2016.1207177.

C2. *The English Indices of Deprivation 2019.* 2019 [cited 2023 Jul 18]; Available from: https://www.gov.uk/government/publications/english-indices-of-deprivation-2019-technical-report

C3. Rue H, Held L. *Gaussian Markov Random Fields*. Chapter 3. 18 February 2005. New York. DOI: https://doi.org/10.1201/9780203492024

C4. Besag, J., York, J. & Mollié, A. *Bayesian image restoration, with two applications in spatial statistics.* Ann Inst Stat Math 43, 1–20 (1991). DOI: https://doi.org/10.1007/BF00116466

C5. Riebler A, Sørbye SH, Simpson D, Rue H. *An intuitive Bayesian spatial model for disease mapping that accounts for scaling.* Statistical Methods in Medical Research. 2016;25(4):1145-1165. DOI: https://doi.org/10.1177/0962280216660421

C6. Burns RA, Butterworth P, Kiely KM, Bielak AAM, Luszcz MA, Mitchell P, et al. 2011. *Multiple imputation was an efficient method for harmonizing the Mini-Mental State Examination with missing item-level data*. Journal of Clinical Epidemiology;64:787-93 doi: 10.1016/j.jclinepi.2010.10.011

Supplementary File E Missing Data – Table E1: Comparison of participants included in our study and those excluded due to missing outcome data

	Participants included in main analysis	Participants excluded from analysis
**Total N**	58,087	7,194
**Ethnicity:**		
**Black African (%)**	2,519 (4.3)	540 (7.5)
**Black Caribbean (%)**	2,197 (3.8)	421 (5.9)
**Indian (%)**	3,153 (5.4)	597 (8.3)
**Pakistani (%)**	2,801 (4.8)	575 (8.0)
**Bangladeshi (%)**	1,584 (2.7)	598 (8.3)
**White (%)**	45,833 (78.9)	4,463 (62.0)
**χ^2 test$^{\pm}$**		p < 0.0001
**Median age in years* (IQR)**	44 (29-60)	41 (28-59)
**Kruskal-Wallis test$^{\pm}$**		p < 0.0001
**Female (%)**	31,168 (53.7)	3,110 (43.2)
**Missing (%)**	0 (0.0)	8 (0.1)
**χ^2 test$^{\pm}$**		p < 0.0001
**UK born (%)**	41,047 (70.7)	4,475 (62.2)
**Missing (%)**	5,898 (10.2)	248 (3.4)
**χ^2 test$^{\pm}$**		p < 0.0001
**Marital status in relationship (%)**	43,387 (74.7)	5,758 (80.0)
**Missing (%)**	52 (0.0)	31 (0.4)
**χ^2 test$^{\pm}$**		p < 0.0001
**Median number of children* (IQR)**	0 (0-1)	0 (0-0)
**Missing (%)**	0 (0.0)	0 (0.0)
**Kruskal-Wallis test$^{\pm}$**		p = 0.0318
**Any health impairment (%)**	18,280 (31.5)	2,067 (28.7)
**Missing (%)**	15 (0.00)	69 (1.0)
**χ^2 test$^{\pm}$**		p < 0.0001
**Urban dwelling (%)**	47,906 (82.5)	6,285 (87.0)
**Missing (%)**	0 (0.0)	0 (0.0)
**χ^2 test$^{\pm}$**		p < 0.0001
**Housing status**		
**Owned (%)**	37,364 (64.3)	4,052 (56.3)
**Private rented (%)**	10,758 (18.5)	1,636 (22.7)
**State rented (%)**	9,701 (16.7)	1,238 (17.2)
**Missing (%)**	264 (0.5)	268 (3.7)
**χ^2 test$^{\pm}$**		p < 0.0001
**Highest education**		
**Postgraduate (%)**	6,052 (10.4)	462 (6.4)
**Undergraduate (%)**	12,827 (22.1)	1,297 (18.0)
**A level (%)**	12,279 (21.1)	1,340 (18.6)
**GCSE (%)**	12,820 (22.1)	1,539 (21.4)
**Other (%)**	5,263 (9.1)	639 (8.9)
**None (%)**	7,514 (12.9)	1,592 (22.2)
**Missing (%)**	1,332 (2.3)	325 (4.5)
**χ^2 test$^{\pm}$**		p < 0.0001
**Employed (%)**	26,177 (45.1)	2,964 (41.2)
**Self-employed (%)**	4,384 (7.6)	644 (9.0)

**Unemployed (%)**	5,632 (9.7)	985 (13.7)
**Retired (%)**	11,495 (19.8)	1,134 (15.8)
**Family leave (%)**	3,720 (6.4)	554 (7.7)
**Student (%)**	6,668 (11.5)	894 (12.4)
**Missing (%)**	11 (0.0)	19 (0.3)
**χ^2 test±**		$p < 0.0001$
**Household monthly income:**		
**6 (highest) (%)**	6,545 (11.3)	1,148 (16.6)
**5 (%)**	4,938 (8.5)	580 (8.4)
**4 (%)**	6,826 (11.8)	611 (8.8)
**3 (%)**	10,393 (17.9)	1,068 (15.5)
**2 (%)**	18,100 (31.2)	1,921 (27.8)
**1 (lowest) (%)**	11,121 (19.2)	1,582 (22.9)
**Missing (%)**	164 (0.3)	284 (3.9)
**χ^2 test±**		$p < 0.0001$
**Index of multiple deprivation decile:**		
**10 (%)(highest)**	4,937 (8.5)	449 (6.2)
**9 (%)**	5,159 (8.9)	539 (7.5)
**8 (%)**	5,325 (9.9)	510 (7.1)
**7 (%)**	5,535 (9.5)	560 (7.8)
**6 (%)**	5,556 (9.6)	558 (7.8)
**5 (%)**	5,613 (9.7)	613 (8.5)
**4 (%)**	5,634 (9.7)	810 (11.3)
**3 (%)**	6,644 (11.4)	1,057 (14.7)
**2 (%)**	7,034 (12.1)	1,159 (16.1)
**1 (lowest) (%)**	6,650 (11.5)	939 (13.1)
**Missing (%)**	0 (0.0)	0 (0.0)
**χ^2 test±**		$p < 0.0001$

* At time of 2014 Immigration Act

± Kruskal-Wallis and χ^2 tests compared characteristics between people included in the study and people excluded from the study

Table E2: Characteristics of participants included in the analysis with complete missing data

	People with complete data	People with missing data[¥]
**Total N (% of all analysis cohort)**	50,585 (87.1)	7,502 (12.9)
**Ethnicity:**		
**Black African (%)**	2,290 (4.5)	229 (3.1)
**Black Caribbean (%)**	2,073 (4.1)	124 (1.7)
**Indian (%)**	2,803 (5.5)	350 (4.7)
**Pakistani (%)**	2,463 (4.9)	338 (4.5)
**Bangladeshi (%)**	1,486 (2.9)	98 (1.3)
**White (%)**	39,470 (78.0)	6,363 (84.8)
**χ^2 test[±]**	Ref	$p < 0.0001$
**Missing**	-	0 (0.0)
**Median age in years* (IQR)**	44 (28-60)	46 (32-63)
**Kruskal-Wallis test[±]**	Ref	$p < 0.0001$
**Missing**	-	0
**Female (%)**	27,125 (53.6)	4,043 (53.9)
**χ^2 test[±]**	Ref	$p = 0.6620$
**Missing**	-	0
**UK born (%)**	40,750 (80.6)	297 (18.5)
**χ^2 test[±]**	Ref	$p < 0.0001$
**Missing**	-	5,898
**Marital status in relationship (%)**	37,796 (74.7)	5,591 (75.0)
**χ^2 test[±]**	Ref	$p = 0.5414$
**Missing**	-	52
**Median number of children* (IQR)**	0 (0-1)	0 (0-1)
**Kruskal-Wallis test[±]**	Ref	$p = 0.1381$
**Missing**	-	0
**Any health impairment (%)**	15,936 (31.5)	2,344 ()
**χ^2 test[±]**	Ref	$p = 0.7335$
**Missing**	-	15
**Urban dwelling (%)**	41,913 (82.9)	5,993 (79.9)
**Missing (%)**	Ref	0
**χ^2 test[±]**	-	$p < 0.0001$
**Housing status**		
**Owned (%)**	32,670 (64.6)	4,781 (65.6)
**Private rented (%)**	9,603 (19.0)	1,115 (15.3)
**State rented (%)**	8,312 (16.4)	1,389 (19.1)
**χ^2 test[±]**	Ref	$p < 0.0001$
**Missing**	-	177
**Highest education**		
**Postgraduate (%)**	5,464 (10.8)	588 (10.7)
**Undergraduate (%)**	11,506 (22.7)	1,321 (22.6)
**A level (%)**	10,703 (21.2)	1,576 (21.6)
**GCSE (%)**	11,450 (22.6)	1,370 (22.6)
**Other (%)**	4,654 (9.2)	609 (9.3)
**None (%)**	6,808 (13.5)	1,332 (13.2)
**χ^2 test[±]**	Ref	$p < 0.0001$
**Missing**	-	11
**Employed (%)**	22,678 (44.8)	3,499 (45.1)
**Self-employed (%)**	3,768 (7.4)	616 (7.5)

**Unemployed (%)**	4,971 (9.8)	661 (9.7)
**Retired (%)**	9,813 (19.4)	1,682 (19.8)
**Family leave (%)**	3,192 (6.3)	528 (6.4)
**Student (%)**	6,163 (12.2)	505 (11.5)
**χ^2 test±**	Ref	p < 0.0001
**Missing**	-	11
**Household monthly income:**		
**6 (highest) (%)**	5,810 (11.5)	735 (11.3)
**5 (%)**	4,324 (8.5)	614 (8.5)
**4 (%)**	5,918 (11.7)	908 (11.8)
**3 (%)**	8,987 (17.8)	1,406 (17.8)
**2 (%)**	15,743 (31.1)	2,357 (31.2)
**1 (lowest) (%)**	9,803 (19.4)	1,318 (19.2)
**χ^2 test±**	Ref	p < 0.0001
**Missing**	-	164
**Index of multiple deprivation decile:**		
**10 (%)(highest)**	4,282 (8.5)	655 (8.7)
**9 (%)**	4,458 (8.8)	701 (9.3)
**8 (%)**	4,563 (9.0)	762 (10.2)
**7 (%)**	4,742 (9.4)	793 (10.6)
**6 (%)**	4,856 (9.6)	700 (9.3)
**5 (%)**	4,859 (9.6)	754 (10.1)
**4 (%)**	4,953 (9.8)	681 (9.1)
**3 (%)**	5,792 (11.5)	852 (11.4)
**2 (%)**	6,319 (12.5)	715 (9.5
**1 (lowest) (%)**	5,761 (11.4)	889 (11.9)
**χ^2 test±**	Ref	p < 0.0001
**Missing**	-	0

* At time of 2014 Immigration Act

± Kruskal-Wallis and χ^2 tests compared characteristics between people included in the study and people excluded from the study

¥ percentages are calculated only from those who have data on the given variable

Table E3: Characteristics of all participants included in analysis cohort, and participants who responded to every exposure period

	Participants who responded to every exposure period	Participants who did not respond to every exposure period
**Total N (% of all analysis cohort)**	21,129 (36.4)	36,958 (63.6)
**Ethnicity:**		
**Black African (%)**	397 (1.9)	2,122 (5.7)
**Black Caribbean (%)**	504 (2.4)	1,693 (4.6)
**Indian (%)**	748 (3.5)	2,405 (6.5)
**Pakistani (%)**	561 (2.7)	2,240 (6.1)
**Bangladeshi (%)**	319 (1.5)	1,265 (3.4)
**White (%)**	18,600 (88.0)	27,233 (73.7)
**Missing (%)**	0 (0.0)	0 (0.0)
**χ^2 test$^{\pm}$**	Ref	p < 0.0001
**Median age in years* (IQR)**	49 (36-63)	40 (26-58)
**Missing (%)**	0 (0.0)	0 (0.0)
**Kruskal-Wallis test$^{\pm}$**	Ref	Ref
**Female (%)**	11,905 (56.3)	19,263 (52.1)
**Missing (%)**	0 (0.0)	0 (0.0)
**χ^2 test$^{\pm}$**	Ref	p < 0.0001
**UK born (%)**	15,316 (72.5)	15,316 (69.6)
**Missing (%)**	3,316 (15.9)	3,316 (7.0)
**χ^2 test$^{\pm}$**	Ref	p < 0.0001
**Marital status in relationship (%)**	15,743 (74.5)	27,644 (74.8)
**Missing (%)**	0 (0.0)	52 (0.1)
**χ^2 test$^{\pm}$**	Ref	p < 0.0001
**Median number of children* (IQR)**	0 (0-1)	0 (0-0)
**Missing (%)**	0 (0.0)	0 (0.0)
**Kruskal-Wallis test$^{\pm}$**	Ref	p < 0.0001
**Any health impairment (%)**	7,150 (33.8)	11,130 (30.1)
**Missing (%)**	0 (0.0)	15 (0.0)
**χ^2 test$^{\pm}$**	Ref	p < 0.0001
**Urban dwelling (%)**	16,595 (78.5)	31,311 (84.7)
**Missing (%)**	0 (0.0)	0 (0.0)
**χ^2 test$^{\pm}$**	Ref	p < 0.0001
**Housing status**		
**Owned (%)**	15,846 (75.0)	21,518 (58.2)
**Private rented (%)**	2,922 (13.8)	7,836 (21.2)
**State rented (%)**	2,334 (11.0)	7,367 (19.9)
**Missing (%)**	27 (0.1)	237 (0.6)
**χ^2 test$^{\pm}$**	Ref	p < 0.0001
**Highest education**		
**Postgraduate (%)**	2,580 (12.2)	3,472 (9.4)
**Undergraduate (%)**	5,754 (27.2)	7,073 (19.1)
**A level (%)**	4,443 (21.0)	7,836 (21.2)
**GCSE (%)**	4,389 (20.8)	8,431 (22.8)
**Other (%)**	1,941 (9.2)	3,322 (9.0)
**None (%)**	1,993 (9.4)	5,521 (14.9)
**Missing (%)**	29 (0.1)	1,303 (3.5)

**χ^2 test±**	Ref	p < 0.0001
**Employed (%)**	10,758 (50.9)	15,419 (41.7)
**Self-employed (%)**	1,713 (8.1)	2,671 (7.2)
**Unemployed (%)**	1,518 (7.2)	4,114 (11.1)
**Retired (%)**	4,862 (23.0)	6,633 (17.9)
**Family leave (%)**	1,221 (5.8)	2,499 (6.8)
**Student (%)**	1,057 (5.0)	5,611 (15.2)
**Missing (%)**	0 (0.0)	11 (0.0)
**χ^2 test±**	Ref	p < 0.0001
**Household monthly income:**		
**6 (highest) (%)**	1,484 (7.0)	5,061 (13.8)
**5 (%)**	2,260 (10.7)	2,678 (7.3)
**4 (%)**	3,375 (16.0)	3,451 (9.4)
**3 (%)**	4,527 (21.4)	5,866 (15.9)
**2 (%)**	6,795 (32.2)	11,305 (30.7)
**1 (lowest) (%)**	2,688 (12.7)	8,433 (22.9)
**Missing (%)**	0 (0.0)	0 (0.0)
**χ^2 test±**	Ref	p < 0.0001
**Index of multiple deprivation decile:**		
**10 (%)(highest)**	2,266 (10.7)	2,671 (7.2)
**9 (%)**	2,277 (10.8)	2,882 (7.8)
**8 (%)**	2,270 (10.7)	3,055 (8.3)
**7 (%)**	2,310 (10.9)	3,225 (8.7)
**6 (%)**	2,198 (10.4)	3,358 (9.1)
**5 (%)**	2,170 (10.3)	3,443 (9.3)
**4 (%)**	1,961 (9.3)	3,673 (9.9)
**3 (%)**	1,951 (9.2)	4,693 (12.7)
**2 (%)**	1,898 (9.0)	5,136 (13.9)
**1 (lowest) (%)**	1,828 (8.7)	4,822 (13.0)
**Missing (%)**	0 (0.0)	0 (0.0)
**χ^2 test±**	Ref	p < 0.0001

Supplementary File F – Sensitivity Analyses: Table F1 – Differences in mean GHQ-12 scores across each exposure period, and according to the number of years since the start of each exposure period, between different ethnic groups following interrupted time series analysis; analysis performed without the use of sample weightings

	Mean difference and credible interval			
	**Exposure period 2 (post Immigration Act 2014)**	**Time since start of exposure period 2 (years)**	**Exposure period 3 (post media coverage 2017)**	**Time since start of exposure period 3 (years)**
**Ethnic group:**				
**Black African** (n = 2,519)	0.43 (-0.22 to 1.07)	-0.24 (-0.55 to 0.06)	0.46 (-0.52 to 1.45)	0.39 (-0.06 to 0.84)
**Black Caribbean** (n = 2,197)	0.68 (0.07 to 1.29)*	-0.18 (-0.47 to 0.11)	1.36 (0.45 to 2.30)*	0.07 (-0.51 to 0.37)
**Indian** (n = 3,153)	0.51 (-0.03 to 1.05)	-0.30 (-0.55 to -0.06)*	0.33 (-0.45 to 1.10)	0.35 (-0.01 to 0.70)
**Pakistani** (n = 2,801)	0.30 (-0.30 to 0.90)	-0.56 (-0.84 to -0.28)*	0.57 (-0.27 to 1.42)	0.26 (-0.11 to 0.64)
**Bangladeshi** (n = 1,584)	-0.41 (-1.21 to 0.39)	-0.23 (0.59 to 0.14)	-0.44 (-1.63 to 0.76)	0.20 (-0.35 to 0.74)
**White** (n = 45,833)	Ref	Ref	Ref	Ref

*Statistically significant result

Table F2 – Differences in mean GHQ-12 scores across each exposure period, and according to the number of years since the start of each exposure period, between different ethnic groups following interrupted time series analysis; complete case analysis, including only individuals without missing data

	**Mean difference and credible interval**			
	**Exposure period 2 (post Immigration Act 2014)**	**Time since start of exposure period 2 (years)**	**Exposure period 3 (post media coverage 2017)**	**Time since start of exposure period 3 (years)**
**Ethnic group:**				
**Black African** (n = 2,290)	0.63 (-0.04 to 1.29)	-0.36 (-0.67 to -0.05)*	0.93 (-0.09 to 1.94)	0.41 (-0.06 to 0.87)
**Black Caribbean** (n = 2,073)	0.68 (0.05 to 1.30)*	-0.13 (-0.45 to 0.16)	1.33 (0.38 to 2.29)*	-0.01 (-0.45 to 0.44)
**Indian** (n = 2,803)	0.38 (-0.18 to 0.94)	-0.20 (-0.46 to 0.06)	0.13 (-0.68 to 0.95)	0.25 (-0.12 to 0.62)
**Pakistani** (n = 2,463)	0.42 (-0.21 to 1.05)	-0.62 (-0.91 to -0.32)*	0.80 (-0.08 to 1.69)	0.34 (-0.06 to 0.73)
**Bangladeshi** (n = 1,486)	-0.46 (-1.27 to 0.36)	-0.23 (-0.60 to 0.15)	-0.75 (-1.97 to 0.48)	0.29 (-0.27 to 0.85)
**White** (n = 39,470)	Ref	Ref	Ref	Ref

*Statistically significant result

Table F3 – Differences in mean GHQ-12 scores across each exposure period, and according to the number of years since the start of each exposure period, between different ethnic groups following interrupted time series analysis; analysis performed including only individuals who responded at least once within each exposure period

	**Mean difference and credible interval**			
	**Exposure period 2 (post Immigration Act 2014)**	**Time since start of exposure period 2 (years)**	**Exposure period 3 (post media coverage 2017)**	**Time since start of exposure period 3 (years)**
**Ethnic group:**				
**Black African** (n = 397)	0.76 (-0.20 to 1.72)	-0.49 (-0.96 to -0.02)*	0.70 (-0.72 to 2.12)	0.89 (0.22 to 1.55)*
**Black Caribbean** (n = 504)	0.64 (-0.16 to 1.44)	-0.11 (-0.50 to 0.28)	1.17 (-0.05 to 2.39)	-0.08 (-0.65 to 0.50)
**Indian** (n = 748)	0.41 (-0.29 to 1.10)	-0.07 (-0.40 to 0.27)	-0.12 (-1.14 to 0.91)	0.35 (-0.13 to 0.83)
**Pakistani** (n = 561)	0.08 (-0.72 to 0.88)	-0.60 (-0.99 to -0.20)*	0.17 (-0.02 to 1.37)	0.44 (-0.10 to 0.98)
**Bangladeshi** (n = 319)	-0.81 (-1.91 to 0.29)	0.10 (-0.43 to 0.63)	-0.59 (-2.22 to 1.03)	-0.32 (-1.08 to 0.44)
**White** (n = 18,600)	Ref	Ref	Ref	Ref

*Statistically significant result
